# Risk of Venous Thromboembolism in People Affected by Charcot Foot Treated With Total Contact Cast

**DOI:** 10.1002/jfa2.70212

**Published:** 2026-09-10

**Authors:** Johan Schoug, Per Katzman, Erik Uddman, Magnus Löndahl

**Affiliations:** ^1^ Department of Endocrinology Skåne University Hospital Lund Sweden; ^2^ Faculty of Medicine The Institution for Clinical Sciences in Lund Lund University Lund Sweden; ^3^ Eli Lilly Sweden AB Helsingborg Sweden

**Keywords:** charcot foot, diabetic foot, immobilization, total contact cast, treatment, venous thromboembolism

## Abstract

**Aims:**

Charcot Foot (CF) is a neuropathy‐induced condition that can lead to fractures and dislocation, warranting long‐time offloading in total contact cast (TCC). It is unknown whether CF or its treatment increases the risk of venous thromboembolism (VTE). This study aims to retrospectively investigate the risk of VTE in people with CF during and after treatment.

**Methods:**

Individuals with diabetes mellitus and active CF treated with TCC at Skåne University Hospital (Lund and Malmö, Sweden) were screened for inclusion in a retrospective cohort study. Baseline characteristics and occurrences of VTE were retrieved using medical records. Causes of death were retrieved from the National Cause of Death Register.

**Results:**

209 individuals with 241 CF episodes were included and followed for 6.8 (3.8–11) years. No VTE was diagnosed during TCC treatment. Two individuals (0.96%) developed VTE after uncasting but before discharge (86–157 days after uncasting; while using boot and orthotic walker, respectively), whereas the other eight individuals (3.8%) had VTE diagnosed after end of CF treatment and follow‐up. Hemodialysis was associated with increased VTE risk after CF diagnosis (HR 13 [3.25–50], *p* < 0.001, accounting for all observed VTE occurrences).

**Conclusion:**

No VTE occurred during TCC treatment, whereas the risk of VTE during the entire CF treatment period was 0.96%.

## 1 Introduction

Charcot's neuroarthropathy or Charcot Foot (CF) is a neuropathy‐induced skeletal condition leading to fractures, joint dislocation and deformity and occurs predominantly as a complication of diabetes. It is managed with offloading of the affected foot, typically using a non‐removable total contact cast (TCC), followed by gradual return of weight‐bearing and walking, using an orthotic walker followed by therapeutic footwear [1]. Initial offloading treatment is long, typically several months, with substantial variation [2, 3, 4] and people affected are at risk of ulceration, need of reconstructive surgery, amputation and increased mortality [3, 5, 6].

Venous thromboembolism (VTE) can occur in states with hypercoagulability, vascular damage, and stasis (Virchow's triad) and is a known complication of immobilization after lower‐limb injury [7, 8]. It is unknown whether CF or its treatment is associated with increased risk of VTE.

The aim of this study is to investigate the incidence of venous thromboembolism in a retrospective Swedish cohort of individuals with CF treated with TCC, during treatment as well as after return of ambulation. We also aimed to examine possible risk factors for VTE in this population.

## 2 Materials and Methods

All adult patients with diabetes mellitus diagnosed with active CF (ICD‐10 codes M90.8H, M90.8, M14.2, M14.6) between 2006–2022 at our tertiary diabetic foot unit were considered for inclusion. Data were retrieved from medical records. Those who had inactive CF or a different diagnosis as assessed by the treating physician, did not have diabetes mellitus, were not primarily treated with a non‐removable TCC, or who rejected study participation were excluded. The date when active CF was suspected and managed at our department was registered as the first visit. Age, sex, diabetes type and duration, HbA1c level, estimated glomerular filtration rate (eGFR), dialysis treatment, concurrent malignancy and regular use of anticoagulant therapy were registered. eGFR was calculated according the revised Lund‐Malmö formula based on creatinine level [9]. Renal impairment was defined as eGFR < 60 mL/min/1.73 m^2^. Biochemistry analyzed as close as possible to the first visit, not exceeding one year, was recorded. Diagnosis and dates of deep‐vein thrombosis (DVT; ICD‐10 I80.1‐9, Z86.7B) and pulmonary embolism (PE; I26.0‐9, Z86.7A) were noted and verified by scrutinizing medical records and radiology reports. Ultrasound examinations were considered to be diagnostic for DVT down to the level of the popliteal vein. Radiology reports from conventional x‐ray and MRI examinations were used to classify CF as stage 0 or 1 (absence or presence of macro‐fractures, joint dislocation, deformity) in accordance with Chantelau and Grützner [10].

Throughout the study period, management of active CF involved initial offloading with TCC until signs of inflammation diminished, a clinical decision which was supported by confirming a local temperature difference < 2°C compared with the corresponding area on the contralateral foot during two sequential visits. In general, during the first weeks, patients changed TCC once weekly, and thereafter every second‐to‐third week. After ending TCC treatment, the general routine included gradually reloading the foot by first transitioning to an orthotic walker, followed by therapeutic boot and shoe (footwear). Clinical follow‐up data and use of offloading modalities (TCC, orthotic walker, boot, and shoe) until December 31st, 2023 were recorded. The full CF treatment period was defined as the time from inclusion to the transition to final footwear or discharge. During the study period, the use of thromboprophylaxis during CF treatment was at the discretion of the treating physician, as no guidelines were established. Causes of death were retrieved from the Swedish National Cause of Death Register (Socialstyrelsen, Stockholm, Sweden). Occurrences of VTE were categorized as follows: during TCC treatment or within 90 days after TCC discontinuation; ≥ 90 days after TCC discontinuation but before transition to final footwear or discharge; after final transition to footwear or discharge.

Categorical data are given as percentages. Continuous data are given as mean (standard deviation) if normally distributed and otherwise as medians and interquartile ranges (IQRs). Shapiro‐Wilk test was used to test for normality. Categorical data were assessed using Fisher's exact test. Continuous data were assessed using the independent samples *t*‐test if normally distributed and otherwise using the Mann–Whitney *U* test. Cox regression was used to estimate proportional hazard ratios (HRs). A *p*‐value < 0.05 was considered statistically significant. Missing cases were excluded pairwise in the analyses. SPSS software (version 30; IBM Corporation, Chicago, IL) was used for statistical analyses.

This study was approved by the Swedish Ethical Review Authority (Dnr 2022‐05609‐01; Uppsala, Sweden).

## 3 Results

**FIGURE 1:**
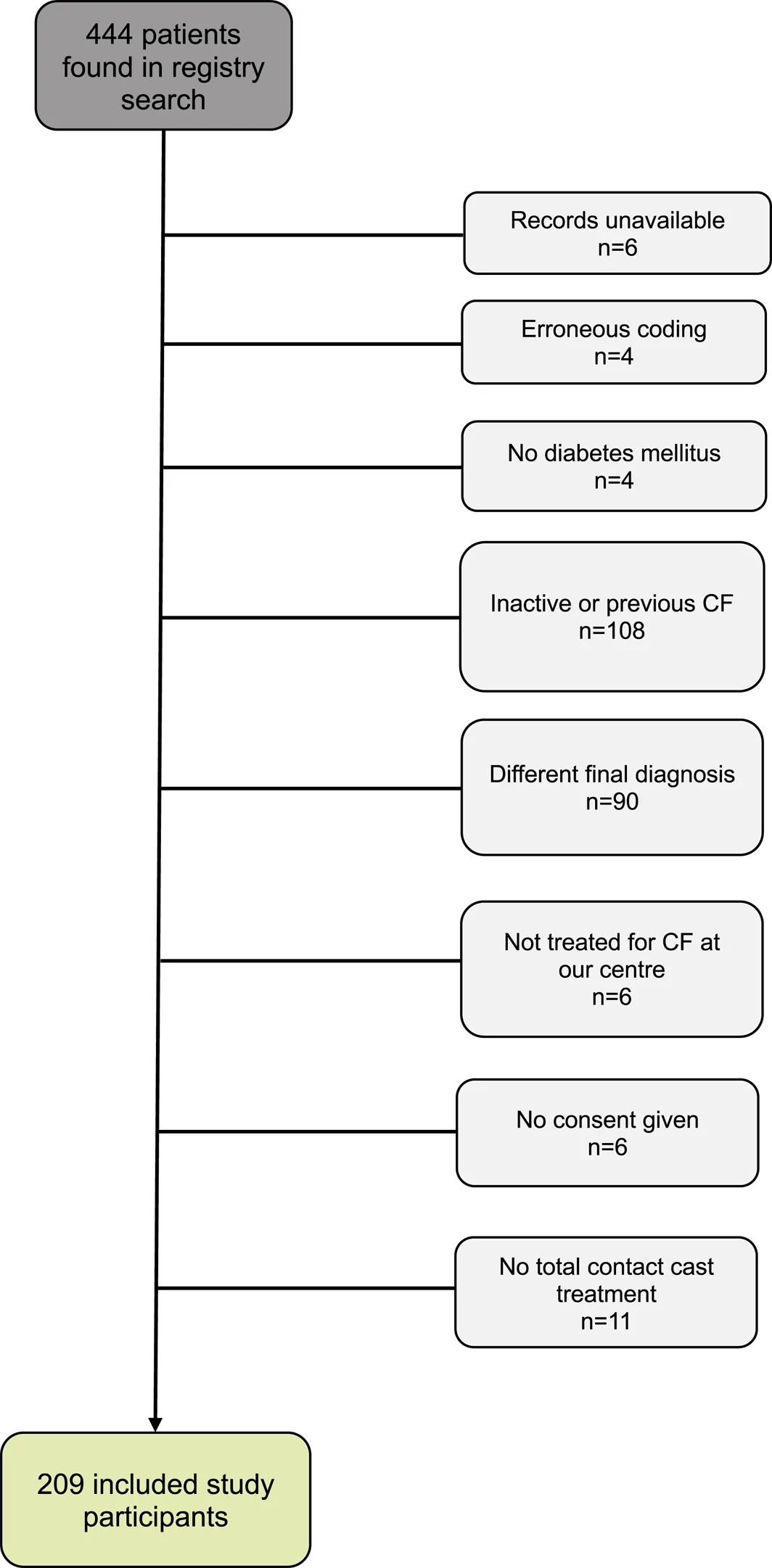
Flow‐chart of the inclusion of individuals in the study.

During the study period, 209 patients, who had 241 CF episodes with initial TCC treatment, were included (Figure 1) and followed for 6.8 (3.8–11) years, that is a total of 1557 patient‐years. The TCC treatment duration was 110 (64.5–175) days, and the full CF treatment duration was 0.94 (0.58–1.45) years. Ten patients (4.8%) were prescribed temporary anticoagulant therapy for thromboprophylaxis during TCC treatment (duration 40 (9–110.5) days), and 27 (13%) were on regular anticoagulant therapy at the time of inclusion.

**FIGURE 2:**
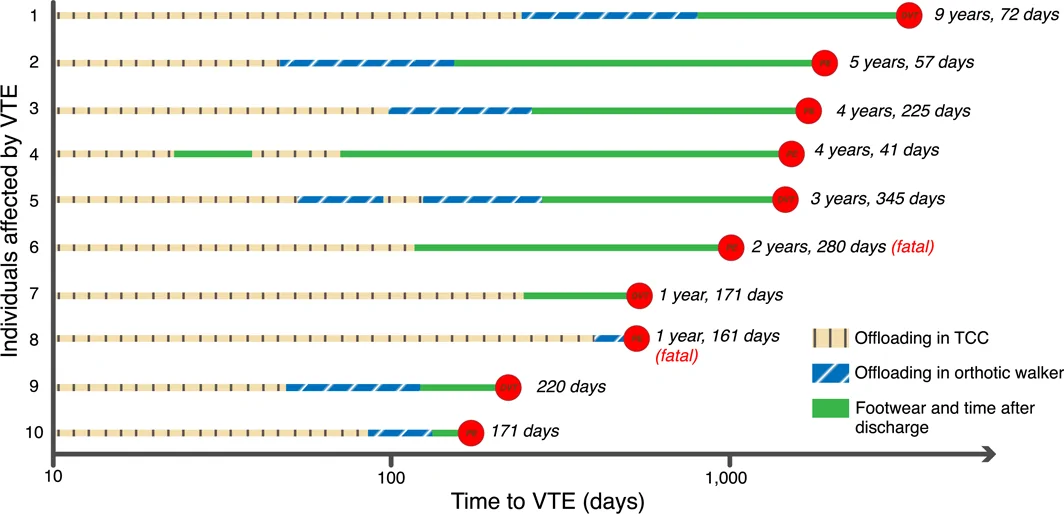
Timeline of the 10 individuals affected by venous thromboembolism (logarithmic scale). DVT, deep‐vein thrombosis, PE, pulmonary embolism; TCC, total contact cast.

During the study period, 10 individuals (4.7%) had a VTE event (Figure 2), corresponding to 6.4 cases of VTE/1000/year. None of these VTE events occurred during treatment with TCC, while one (0.48%) occurred 86 days after uncasting during use of boot, and another occurred 157 days after uncasting during use of orthotic walker (together comprising 0.96% of all individuals; 1.1% among individuals not on regular anticoagulants). Both individuals had PE. Their preceding TCC duration was 85–396 days, respectively. Both these individuals were males in the 6th–7th decades of life, had type 1 diabetes, had stage 1 CF, and were on hemodialysis treatment, and one was diagnosed with metastasized cancer soon after the VTE event. The other eight individuals had VTE after end of CF follow‐up (median 1373.5; IQR 443–1771; range 171–3117 days after final uncasting). Their previous total TCC duration was 90 (50.5–211; range 47–245) days. Six of the 10 VTE occurrences during the whole study period were PE, with two being fatal. The four DVT were diagnosed at the level of the iliacal vein, femoral vein (two cases), and popliteal vein, respectively.

Patient characteristics are given in Table 1. Individuals on hemodialysis treatment at baseline had a significantly higher risk of VTE (HR 13 [3.25–50], *p* < 0.001, based on all VTE occurrences during the study period). Additionally, another patient who started dialysis treatment after onset of CF had VTE. After excluding individuals with regular anticoagulant use, hazard ratio for cerebrovascular disease and eGFR were statistically significant (5.50 [1.14–27], *p* = 0.03; 0.98 per mL/min/1.73 m^2^ [0.95–1.00], *p* = 0.046). VTE risk (based on all VTE occurrences) was 4.5% in those treated prophylactically with anticoagulants during TCC treatment versus 5.0% in those not (HR 1.09 [0.14–8.8], *p* = 0.93).

**TABLE 1:** Descriptive statistics and univariable analysis of risk factors for VTE.

	All patients (*n* = 209)	VTE at any time (within category)	VTE at any time (in others)	Hazard ratio (95% CI)	*p*
Age (years)	60 (12)			1.009 (0.96–1.06)	0.75
Male sex	132 (63%)	6 (4.5%)	4 (5.2%)	0.93 (0.26–3.30)	0.91
Smoking history (ever)	73 (35%)	2 (2.7%)	8 (5.9%)	0.52 (0.11–2.46)	0.41
Type 1 diabetes	73 (35%)	6 (8.2%)	4 (2.9%)	0.41 (0.11–1.45)	0.16
Diabetes duration (years)	17 (10–28)			0.98 (0.93–1.03)	0.41
eGFR (mL/min/1.73 m^2^)	68 (44.5–84.5)			0.98 (0.96–1.003)	0.08
eGFR < 60 mL/min/1.73 m^2^	84 (41%)	5 (6.0%)	5 (4.1%)	1.73 (0.50–6.03)	0.39
Hemodialysis	9 (4.3%)	3 (33%)	7 (3.5%)	13 (3.25–50)	< 0.001
HbA1c (%)	8.0 (6.9–9.3)			1.05 (0.73–1.49)	0.80
HbA1c (mmol/mol)	64 (52–78)			1.004 (0.97–1.04)	0.80
HbA1c > 70 mmol/mol (> 8.6%)	72 (37%)	5 (6.9%)	4 (3.2%)	2.17 (0.58–8.09)	0.25
Ischemic heart disease	31 (15%)	2 (6.5%)	8 (4.5%)	1.67 (0.35–7.90)	0.52
Cerebrovascular disease	15 (7.2%)	2 (13%)	8 (4.1%)	3.50 (0.74–17)	0.11
Systolic heart failure	9 (4.3%)	1 (11%)	9 (4.5%)	3.62 (0.45–29)	0.23
Peripheral arterial disease	42 (20%)	2 (4.8%)	8 (4.2%)	1.13 (0.24–5.34)	0.88
Malignancy	7 (3.3%)	1 (14%)	9 (4.5%)	4.76 (0.60–38)	0.14
Previous VTE	10 (4.8%)	1 (10%)	9 (4.5%)	3.31 (0.41–26)	0.26
Regular anticoagulant treatment	26 (13%)	1 (3.8%)	9 (5.0%)	0.96 (0.12–7.61)	0.97
Prophylactic anticoagulant during CF treatment^a^	22 (12%)	1 (4.5%)	8 (5.0%)	1.09 (0.14–8.78)	0.93
Total TCC duration (days)	114 (69–176.5)			1.00 (0.99–1.01)	0.98
Stage 1 CF	132 (63%)	6 (4.5%)	4 (5.2%)	0.96 (0.27–3.40)	0.95

*Note:* Descriptive statistics and univariable analysis of risk factors for VTE. Four individuals had missing values for eGFR, 13 individuals had missing values for HbA1c, and two had missing data regarding anticoagulation.

Abbreviations: CF, Charcot Foot; TCC, total contact cast; VTE, venous thromboembolism.

^a^ Only patients without chronic anticoagulant treatment were included.

## 4 Discussion

In a cohort of 209 individuals treated for CF with TCC for a mean duration of more than three and a half months, no case of VTE was diagnosed during TCC treatment and one VTE (0.48%) occurred within 90 days after uncasting. Although the number of individuals is limited, this suggests that the risk of VTE during TCC in people with CF is similar to the rate of 0.5%–0.85% reported in other studies of immobilization due to lower‐limb injury [7, 8]. Further, in a retrospective study including 136 casting episodes in patients with neuropathic foot ulcers or CF, Tonge et al. reported a low VTE incidence. In their study, with a shorter casting period (mean duration 45 days) and a slightly younger study population (mean age 55 years) compared to our study, the only VTE occurred in an immobilized 91 years old man with several established risk factors for VTE, including active malignancy [11].

Of the 10 VTE events in our study, two events occurred after uncasting while under management of management of inactive CF. Both individuals were treated with hemodialysis, which is recognized as a risk factor for VTE in other reports [12, 13]. It is unclear whether active CF and its treatment per se increased the risk of VTE. Treatment with TCC limits mobility, immobilizes the ankle and redistributes weight‐bearing from foot to leg, but does not inhibit walking, and the reduction of mobility probably depends on both habitual physical ability and compliance. Later during the treatment course, gradual return of ambulation commences, and reduced ambulation in this stage of the treatment course as well as preceding TCC treatment may have contributed to an increased VTE risk in these two cases, in combination with other risk factors. Although it is unclear if thromboprophylaxis should be given to individuals with CF, our results suggest considering it mainly for those with multiple VTE risk factors, such as malignancy, hemodialysis, advanced age, or a history of VTE and coagulation disorders. Most cases (*n* = 8) of VTE in this cohort occurred after completion of CF treatment and follow‐up. The total incidence in this cohort was much higher than reported incidence for pulmonary embolism [14] and deep‐vein thrombosis [15] in the general population in Sweden. This suggests individuals affected by CF more often are at risk of VTE, although no control group with individuals with DM but not CF was available, limiting the possibility to assess the impact CF had on VTE risk in these individuals.

Although including a well‐characterized clinical CF population, the main weakness of our study is the limited number of patients and VTE events, relative to the study aim. However, as treatment periods in general are long, the total number of days on TCC was, in relative terms, high. No control group was used, which makes the VTE risk in this cohort more difficult to interpret. The methodology relies on ICD‐10 diagnosis coding, possibly leading to CF and VTE cases not being identified. However, both CF and VTE are managed with follow‐up visits to specialist departments in the entire Skåne region, each documented with a diagnosis code, making such misses less likely. However, some individuals may have moved outside the catchment area and had VTE not recognized later during the study period. Heterogeneity between individual CF cases is introduced from varying TCC treatment duration and the lack of uniform criteria for identifying active CF and its remission. However, this is inherently the case, due to the way CF has been and continues to be managed [1]. Finally, the retrospective nature of this study confers an inherent risk of bias.

## 5 Conclusion

In conclusion, this study finds that VTE during CF treatment occurred in two individuals (0.96%), after completion of TCC treatment and in individuals with established risk factors for VTE. Individuals affected by CF seem to be at risk of VTE after end of CF treatment. We suggest that future prospective studies of CF should include VTE as an adverse outcome of interest.

## Author Contributions

**Johan Schoug:** conceptualization, data curation, formal analysis, investigation, methodology, visualization, writing – original draft, writing – review and editing. **Per Katzman:** writing – review and editing. **Erik Uddman:** writing – review and editing. **Magnus Löndahl:** funding acquisition, methodology, writing – review and editing.

## Disclosure

M.L. is an employee of Eli Lilly and Company. E.U. is on the speakers' list for Eli Lilly and Company. J.S. has nothing to disclose. P.K. has nothing to disclose.

## Ethics Statement

This study was approved by the Swedish Ethical Review Authority (Dnr 2022‐05609‐01; Uppsala, Sweden).

## Conflicts of Interest

The authors declare no conflicts of interest.

## Data Availability

Data analyzed in the current study are available from the corresponding author upon reasonable request.
